# Does a mobile laminar airflow screen reduce bacterial contamination in the operating room? A numerical study using computational fluid dynamics technique

**DOI:** 10.1186/1754-9493-8-27

**Published:** 2014-06-26

**Authors:** Sasan Sadrizadeh, Ann Tammelin, Peter V Nielsen, Sture Holmberg

**Affiliations:** 1 Division of Fluid and Climate Technology, School of Architecture and the Built Environment, KTH Royal Institute of Technology, Stockholm, Sweden; 2 Department of Medicine, Solna (MedS), Unit of Infectious Diseases, Karolinska Institutet, Stockholm, Sweden; 3 Department of Civil Engineering, Aalborg University, Aalborg, Denmark

**Keywords:** Air-borne bacteria, Colony-forming unit, Operating room, Microbiological air quality, Mobile ultraclean exponential laminar airflow screen, CFD simulation

## Abstract

**Background:**

Air-borne bacteria in the operating room (OR) may contaminate the surgical wound, either by direct sedimentation from the air or indirectly, by contaminated sterile instruments. Reduced air contamination can be achieved with an efficient ventilation system. The current study assesses the additive effect of a mobile laminar airflow (MLAF) unit on the microbiological air quality in an OR supplied with turbulent-mixing air ventilation.

**Methods:**

A recently designed OR in NKS (Nya Karolinska Sjukhuset, Stockholm, Sweden) was the physical model for this study. Simulation was made with MLAF units adjacent to the operating table and the instrument tables, in addition to conventional turbulent-mixing ventilation. The evaluation used numerical calculation by computational fluid dynamics (CFD). Sedimentation rates (CFU/m^2^/h) were calculated above the operating table and two instrument tables, and in the periphery of the OR. Bacterial air contamination (CFU/m^3^) was simulated above the surgical and instrument tables with and without the MLAF unit.

**Results:**

The counts of airborne and sedimenting, bacteria-carrying particles downstream of the surgical team were reduced to an acceptable level for orthopedic/implant surgery when the MLAF units were added to conventional OR ventilation. No significant differences in mean sedimentation rates were found in the periphery of the OR.

**Conclusions:**

The MLAF screen unit can be a suitable option when the main OR ventilation system is unable to reduce the level of microbial contamination to an acceptable level for orthopedic implant surgery. However, MLAF effect is limited to an area within 1 m from the screen. Increasing air velocity from the MLAF above 0.4 m/s does not increase the impact area.

## Background

### Surgical site infections, air-borne transmission and ventilation

Surgical-site infections (SSIs) are serious and contribute to higher rates of patient morbidity and mortality, increased hospitalization time, and patient dissatisfaction [1]. Infections after hip- and knee-prosthetic surgery are devastating. Several measures must be taken to reduce the infection rate [2].

It is well-known that operating room (OR) personnel are the main source of airborne bacteria as they disseminate infectious particles into their surrounding environment. A person releases about 10^4^ skin scales per minute during walking, 10 percent of which carry bacteria [3]. However, the count of discharged microorganisms varies widely, even as much as 12-fold, between individuals and sampling days [4]. The size of the particles carrying microorganisms has been reported ranging from 4–60 μm [5,6]. Bacteria suspended in the OR air may contaminate the surgical wound, either by direct sedimentation from the air or indirectly by contaminated surgical instruments [7].

Air contamination can be reduced with an efficient ventilation system to dilute and evacuate contaminants from the OR [8], increasing the performance of staff clothing to prevent bacteria shedding to the air [9], and restricting the number of people and their activity in the OR [10,11].

Laminar airflow (LAF) is the most efficient OR ventilation system [8,12]. However, indoor obstacles including medical lamps, surgical staff, and equipment can easily affect the unidirectional airflow pattern of a vertical LAF system [13,14]. Intended colony-forming unit (CFU) levels (<10 CFU/m^3^) will not be present, and the desired SSI-rate decrease cannot be achieved. Installing a LAF ventilation system might also be difficult in existing ORs and is costly when constructing new ORs. A mobile laminar airflow (MLAF) unit could overcome both the problem with physical obstacles and costs.

A MLAF unit with conventional turbulent-mixing ventilation is a valuable complement to general ventilation in reducing bacterial load during operations in an OR [15-19]. The authors conclude that the additional MLAF screen reduced the number of viable airborne bacteria and sedimenting, bacteria-carrying particles (BCPs) to the same level as ultra-clean LAF-ventilation.

### Microbiological air-sampling methods

Currently, microbiological air sampling in ORs is performed either by passive air sampling (PAS) with settle plates or by active air sampling (AAS) with a slit sampler, impaction sampler, or filter sampler [20]. PAS measures the settlement rate of viable particles on surfaces, while AAS provides information about the concentration of viable particles in the air. Both methods require access to a bacteriological laboratory and can only be used in existing ORs. Sadrizadeh et al. provides a more detailed explanation of the mathematical modeling of active and air-sampling methods [10].

### Computational fluid dynamics

It is important to control air distribution in enclosed spaces to create and maintain a comfortable, healthy atmosphere for occupants, especially in sensitive indoor environments such as ORs. Experimental studies provide direct evidence of airflow and particle-transport phenomena. However, the complexity of indoor airflow makes experimental investigation very difficult and expensive.

With recent advances in computer technology in various methods, computational fluid dynamics (CFD) has become an essential complementary tool to physical experiments. CFD is the science of predicting fluid or gas flow, which largely reduces the number of required physical experiments and provide great potential for improving prediction accuracy of air distribution in enclosed environments. This method was successfully applied in simulated OR environments [10,13,21]. The data made it possible to clarify uncertainties at initial stages of design. Obtaining precise information can provide an important foundation on which to base design decisions. It can also overcome some measurement limitations and extend the range of research. Generally, the CFD technique has three main steps.

***Pre-processing:*** This step consists of defining simulation domain and grid generation. The domain in which flow is to be analyzed requires modeling, generally with a CAD software package. For the complex geometries, some degree of simplification may be required to correct the geometry and make it valid as a CFD model. Portions of the flow-domain boundary coincide with the surfaces of the body geometry. The spatial geometric spaces the fluid occupies are modeled so as to provide input for grid generation. Mesh generation is essential in the CFD analysis process, which subdivides the domain into discrete cells, known as *grid* or *mesh*. The created mesh surrounds the object and then extends in all directions to get the physical properties of the surrounding fluid; in other words, the OR air in the present study. The mesh is very fine in areas with large gradients in the flow field and coarser in regions with relatively little change.

***Boundary condition and solve:*** Numerical simulation generally requires input parameters consisted of the desired strategy. The boundary conditions are specified as the fluid properties and behavior at the boundaries of the problem, inlet temperature and velocity, and particle generation rate. The numerical solution is obtained by an iterative method, which achieves high accuracy using a large number of repetitions. As the simulation proceeds, the solution is monitored to determine if a converged solution has been obtained.

***Post-processing:*** This stage involves extracting the desired flow properties (velocity, particle concentration, temperature) from the computed flow field. This is accomplished by means of contour and color plots, vector plots, and animation for dynamic result.

Result sensitivity should be examined to understand possible differences in the accuracy of results and with respect to initial flow conditions and experimental investigation.

## Methods

**Figure 1:**
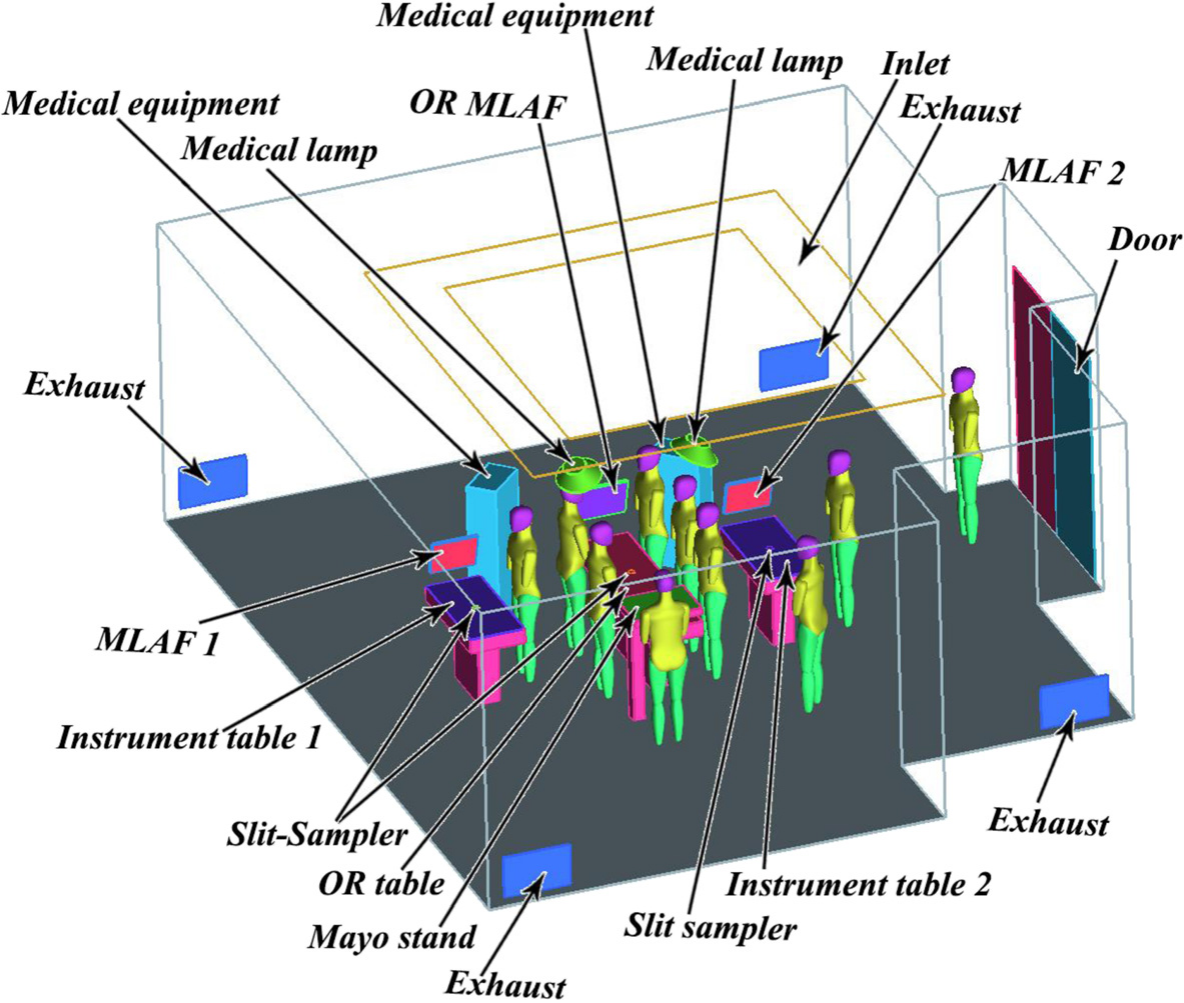
An isometric view of the OR model.

A newly designed NKS (Nya Karolinska Sjukhuset) OR, which was adopted in the authors’ previous work, [10,22] was chosen as the physical model for this study. The OR dimensions are L 8.5 m × W 7.7 m× H 3.2 m. Figure 1 shows the geometrical configuration.

**Figure 2:**
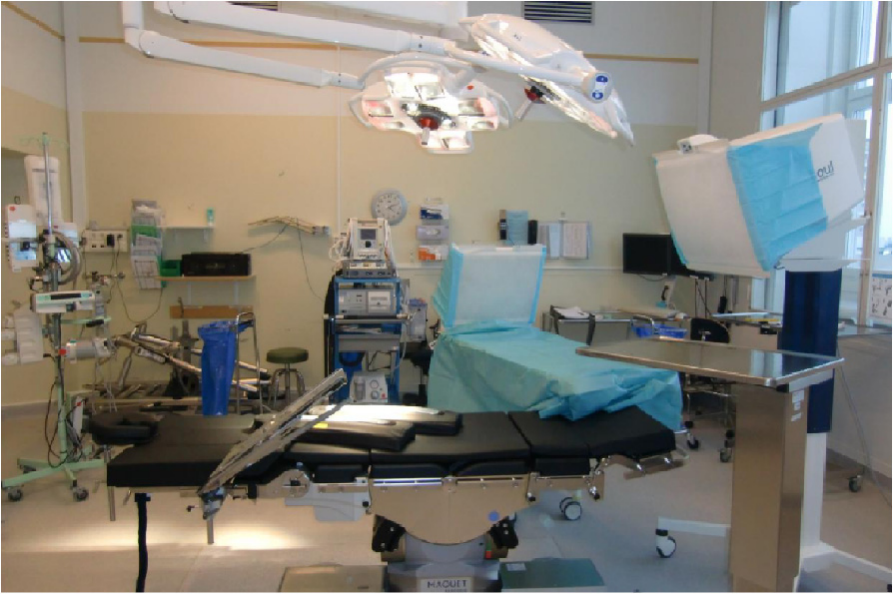
Two MLAF units in an OR. Photo from South Hospital (Södersjukhuset), Stockholm.

Ventilating air was introduced through 24 diffusers, evenly spaced over the ceiling, with a total airflow rate of 2500 L/s, yielding a design ventilation rate 47 air changes per hour (ACH). Outgoing air was extracted through four exhaust openings placed on the parallel vertical walls at floor level. Ten surgical staff members were placed in upright stationary positions, mostly around the operating table. A MLAF (TOUL-400) screen was placed at the foot end of the operating table, with the air flow directed along the table. Two sterile instrument tables equipped with MLAFs (TOUL-300) were also considered. Figure 2 shows two MLAF units in an OR, one placed at the foot of the operating table and the other at the end of an instrument table. A source strength (mean value of CFUs emitted from one person per second) of five CFU/s per individual was considered for each surgical staff member, in accordance with SIS-TS 2012 [23]. Simulation was performed under different MLAF centerline velocities from zero (MLAF unit switched off) to 1.0 m/s.

The authors performed a comprehensive validation of the flow field between numerical CFD results and measured data [10]. The relative error between measurement and simulation was less than 5 percent. However, this aspect was outside the scope of the present study and not analyzed here.

In this study, both AAS and PAS approaches were numerically simulated. The simulated AAS method estimated air at a total flow rate of 100 L/min, drawn for 10 min through a slit sampler installed above each table. The total number of CFU was then counted, and the concentration presented as the number of CFU/m^3^. This gave a direct quantitative estimate of the number of CFUs in the sampled air. In the simulated PAS approach, the total surface area of the tables were exposed to the OR air during one hour, and results were estimated as CFU/m^2^/h. For each case, air sampling was repeated 100 times to achieve statistically reliable results. Sadrizadeh et al. provide more details of numerical AAS and PAS calculations [10,22].

## Results

Table 1 shows the mean value of volumetric BCP concentration (CFU/m^3^) as a function of MLAF screen velocity obtained by CFD simulation of the AAS method. Mean concentration of BCPs in the air above the operating table was 19 CFU/m^3^ with standard OR ventilation. When the MLAF unit was functioning at a velocity of 0.4 m/s, BCP concentrations decreased to a mean value of 1 CFU/m^3^. The same BCP concentration trend was seen for instrument table one. The number of microorganisms in the air above instrument table two was lower with standard OR ventilation than for the other tables, due to the local airflow pattern and the distance of this table from the surgical team members. However, when the MLAF unit was functioning, the concentration of 8 CFU/m^3^ dropped to a concentration of 1 CFU/m^3^.

**Table 1:** Mean values of volumetric bacteria-carrying particles for different centerline velocity of the mobile laminar screen unit

**MLAF screen velocity**	**Operating table**	**Instrument table one**	**Instrument table two**
	**CFU/m**^**3**^	**CFU/m**^**3**^	**CFU/m**^**3**^
**(m/s)**	**Mean (min–max)**	**Mean (min–max)**	**Mean (min–max)**
off	19.08 (9–32)	18.12 (13–28)	7.66 (5–24)
0.2	9.94 (4–12)	8.36 (3–13)	1.82 (1–4)
0.4	1.21 (1–4)	1.14 (0–5)	0.90 (0–3)
0.6	0.72 (0–3)	0.08 (0–1)	0.08 (0–1)
0.8	0.04 (0–1)	0.06 (0–1)	0.00 (0–0)
1.0	0.00 (0–0)	0.00 (0–0)	0.00 (0–0)

CFD simulation results were based on active air-sampling method. (CFU: colony-forming unit, MLAF: mobile laminar airflow).

**Figure 3:**
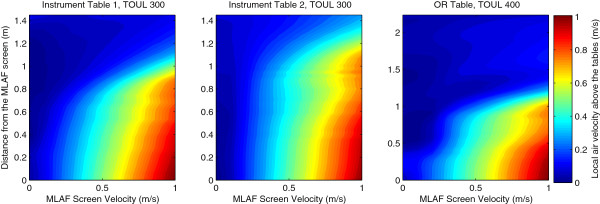
MLAF impact area as a function of screen-unit velocity and distance from the MLAF screen center.

Table 2 shows the mean value of BCP sedimentation rate (CFU/m^2^/h) above the tables and in the OR periphery, with and without a MLAF screen, when simulating the PAS method. When the main OR ventilation system functioned without a MLAF screen, the mean values of BCP sediment distribution on the surface area of the operating table, and instrument tables one and two, respectively were 180, 225, and 51 CFU/m^2^/h. When the MLAF unit was at a velocity of 0.4 m/s, the sedimentation rates were reduced to values of 14, 37, and 19 CFU/m^2^/h. There was no further reduction when the velocity increased from 0.4 m/s to 1.0 m/s. No major difference in BCP sedimentation rate could be observed in the peripheral area of the OR, with or without MLAF units.Figure 3 shows the impact area of MLAF units, as a function of screen velocity and the distance from the center point of the MLAF screen. The area impact of MLAF greatly depends on screen velocity. In this case, a screen velocity of 0.4 m/s already gave good particle elimination at a distance of 1 m away from the screen.

**Table 2:** Mean values of sedimenting bacteria-carrying particles for different centerline velocities of the MLAF unit

**MLAF screen velocity**	**Operating table**	**Instrument table one**	**Instrument table two**	**OR periphery**
	**CFU/m**^**2**^**/h**	**CFU/m**^**2**^**/h**	**CFU/m**^**2**^**/h**	**CFU/m**^**2**^**/h**
**(m/s)**	**Mean (min–max)**	**Mean (min–max)**	**Mean (min–max)**	**Mean (min–max)**
off	180.78 (157–210)	225.80 (155–312)	51.06 (32–63)	382.85 (289–401)
0.2	43.94 (34–55)	96.44 (83–121)	27.86 (23–35)	380.36 (254–421)
0.4	14.02 (11–20)	37.42 (32–65)	19.92 (14–29)	393.69 (298–501)
0.6	15.84 (8–19)	29.54 (21–52)	18.80 (15–24)	390.50 (301–423)
0.8	14.70 (9–14)	22.72 (21–40)	17.44 (13–22)	384.85 (319–419)
1.0	14.60 (8–14)	17.32 (12–38)	14.46 (14–21)	386.07 (329–438)

CFD simulation results were based on the passive air-sampling method. (CFU: colony-forming unit, MLAF: mobile laminar airflow).

## Discussion

The results from the CFD simulation show that MLAF can play a marked role in reducing microbial contamination of the critical surgical zone. CFU counts showed obvious declining trends when the MLAF airflow functioned, both when analyzed as CFU/m^3^ and CFU/m^2^/h. Surgeries in which air-borne transmission of microorganisms should be reduced to a minimum (such as hip- and knee-prosthetic surgery) can be safely performed in the examined OR with the current number of staff members, even if the MLAF screen functions at the minimum velocity of 0.4 m/s. The desired count of < 10 CFU/m^3^ for infection-sensitive surgery was reached at that velocity.

Friberg et al. [15] evaluated the MLAF screen unit and found that at a distance of 1.4 m–1.6 m away from the screen, the bacterial count reduction rate was moderate, at approximately 80 percent. CFU simulation showed that an increase of MLAF air velocity above 0.4 m/s did not increase the impact area. Enough washing effect to remove pathogens before they settle can be obtained at a distance of about 1 m away from the screen, but not further. This shows that optimal positioning of the screen unit was critical for removal efficiency. It also indicated a limitation of the usefulness of a MLAF unit.

## Conclusion

The MLAF screen unit can be a suitable option when the main OR ventilation system is unable to reduce the level of microbial contamination to an acceptable level for orthopedic implant surgery. However, MLAF effect is limited to an area within 1 m from the screen. An increase of MALF air velocity above 0.4 m/s does not significantly increase the impact area.

It would be valuable to perform the same simulation with and without MLAF with the staff dressed in a clothing system resulting in a source strength of 1.5 CFU/s–2.5 CFU/s per person [9,24].

## Abbreviations

MLAF: Mobile laminar airflow; CFD: Computational fluid dynamics; OR: Operating room; SSI: Surgical-site infections; LAF: Laminar airflow; CFU: Colony-forming unit; BCP: Bacteria-carrying particles; PAS: Passive air sampling; AAS: Active air samplers; ACH: Air changes per hour.

## Competing interests

The authors declare they have no competing interests with regard to this publication.

## Authors’ contributions

SS: Primary author, who performed the simulation and drafted the manuscript. AN: Provided advice on the study’s methodology, interpretation of the data and helped revise and refine the final manuscript. PVN: Critically edited and assisted in manuscript authorship. SH: Coordinated the study and helped to draft the manuscript. All authors read and approved the final manuscript.
